# Probiotic Intake and Inflammation in Patients With Chronic Kidney Disease: An Analysis of the CKD-REIN Cohort

**DOI:** 10.3389/fnut.2022.772596

**Published:** 2022-03-30

**Authors:** Sandra Wagner, Thomas Merkling, Marie Metzger, Laetitia Koppe, Maurice Laville, Marie-Christine Boutron-Ruault, Luc Frimat, Christian Combe, Ziad A. Massy, Bénédicte Stengel, Denis Fouque

**Affiliations:** ^1^Université de Lorraine, INSERM CIC 1433, Nancy CHRU, Inserm U1116, Nancy, France; ^2^FCRIN INI-CRCT, Nancy, France; ^3^Centre de Recherche en Epidémiologie et Santé des Populations (CESP), Univ Paris-Saclay, UVSQ, INSERM, Equipe Epidémiologie Clinique, Villejuif, France; ^4^Département de Néphrologie, Hopital Lyon Sud – HCL, Pierre-Bénite, France; ^5^Université de Lyon, CarMeN Laboratory, INSA-Lyon, INSERM U1060, INRA, Université Claude Bernard Lyon 1, Villeurbanne, France; ^6^Centre de Recherche en Epidémiologie et Santé des Populations (CESP), Université Paris-Saclay, UVSQ, INSERM, Gustave Roussy, Equipe Exposome-Hérédité, Villejuif, France; ^7^EA4360 APEMAC, Université de Lorraine, Université Paris Descartes, Nancy, France; ^8^Département de Néphrologie, CHU de Nancy, Vandoeuvre-lès-Nancy, France; ^9^Service de Néphrologie-Transplantation-Dialyse-Aphérèse, Centre Hospitalier Universitaire de Bordeaux, Bordeaux, France; ^10^INSERM, U1026, Université Bordeaux Segalen, Bordeaux, France; ^11^Service de Néphrologie, Hôpital Ambroise Paré, APHP, Paris, France

**Keywords:** inflammation, C-reactive protein, yoghurt, probiotic, chronic kidney disease, epidemiology

## Abstract

**Background and Aims:**

Little is known about the effects of probiotics on inflammation in the context of chronic kidney disease (CKD). We investigated the association between probiotic intake and inflammation in patients with moderate-to-advanced CKD.

**Methods:**

We performed a cross-sectional study of 888 patients with stage 3–5 CKD and data on serum C-reactive protein (CRP) levels and a concomitant food frequency questionnaire. We estimated the odds ratios (ORs) [95% confidence interval (CI)] for various CRP thresholds (>3, >4, >5, >6, and >7 mg/L) associated with three intake categories (no yoghurt, ordinary yoghurt, and probiotics from yoghurts or dietary supplements) and two frequency categories (daily or less than daily).

**Results:**

The 888 study participants (median age: 70; men: 65%) had a median estimated glomerular filtration rate of 28.6 mL/min/1.73 m^2^ and a median [interquartile range] CRP level of 3.0 [1.6, 7.0] mg/L. Fifty-seven percent consumed ordinary yoghurt and 30% consumed probiotic yoghurt. The median intake frequency for yoghurt and probiotics was 7 per week. Relative to participants not consuming yoghurt, the ORs [95% CI] for CRP > 6 or >7 mg/L were significantly lower for participants consuming ordinary yoghurt (0.58 [0.37, 0.93] and 0.57 [0.35, 0.91], respectively) and for participants consuming probiotics (0.54 [0.33, 0.9] and 0.48 [0.28, 0.81], respectively), independently of age, sex, body mass index, CKD stage, cardiovascular disease, and fibre, protein and total energy intakes. The ORs were not significantly lower for CRP thresholds >3, >4, and >5 mg/L and were not significantly greater in daily consumers than in occasional consumers.

**Conclusion:**

We observed independent associations between the consumption of yoghurt or probiotics and lower levels of inflammation in patients with CKD. There was no evidence of a dose-effect relationship.

**Clinical Trial Registration:**

[https://www.clinicaltrials.gov/ct2/show/NCT03381950], identifier [NCT03381950].

## Introduction

Inflammation is a prominent feature of chronic kidney disease (CKD), which affects 10–15% of the population worldwide (1–4). Furthermore, inflammation is an established risk factor for early mortality and acts as a catalyst for the development of other complications, such as cardiovascular disease (CVD) (5). Although inflammation has many causes, gut dysbiosis (i.e., alteration of the intestinal microbiota) is now emerging as a key topic of interest (2, 6, 7).

Probiotic consumption appears to be a promising means of limiting dysbiosis and its harmful effects on health (8–10). Probiotics have been defined by the United Nations’ Food and Agriculture Organization (FAO) and the World Health Organization (WHO) as “live microorganisms that, when administrated in adequate amounts, confer a health benefit on the host” (11). Probiotics can modulate the composition of the intestinal microbiota, create a more favourable environment, and in principle reduce inflammation. Only a few clinical trials have focused on the effects of probiotics (e.g., dietary supplements) on inflammation in CKD, and the results have been inconsistent (12–17). In some studies of patients on dialysis, probiotic intake was associated with lower levels of pro-inflammatory cytokines, such as Tumour Necrosis Factor-α (TNF-α), Interleukins (IL-6, and IL-5), or C-reactive protein (CRP) and higher levels of anti-inflammatory cytokines (e.g., IL-10) (12–15). In contrast, other studies did not find any effects of probiotics on inflammatory markers (16, 17).

Although probiotic microorganisms can be specifically included in the diet as supplements, they are naturally present in food items such as yoghurts. The commercial use of the denomination “yoghurt” is strictly regulated: according to the FAO/WHO Codex Alimentarius, the milk must have been fermented by only two authorised bacterial strains (*Lactobacillus delbrueckii* subsp. *bulgaricus* and *Streptococcus salivarius* subsp. *thermophilus*), and the finished product must contain at least 10 million living bacterial organisms per g. The National Health and Nutrition Examination Survey (NHANES) investigated the association between probiotics/yoghurt intake and CKD; frequent intake was found to be associated with a lower likelihood of proteinuria (18). The NHANES did not, however, investigate levels of inflammation markers.

The primary objective of the present study was to investigate the associations of the intake of probiotic-containing yoghurt/dietary supplements or ordinary yoghurt with inflammation (as assessed by the serum CRP concentration) in patients with CKD stages 3–5. We also investigated the association between the frequency of intake and inflammation. We hypothesised that both probiotic intake and ordinary yoghurt intake would be associated with lower levels of inflammation.

## Population and Methods

The CKD-Renal Epidemiology and Information Network (CKD-REIN) study is a prospective cohort study carried out in 40 private- or public-sector nephrology clinics throughout France. Between July 2013 and March 2016, the study included 3033 adult outpatients with moderate or advanced CKD [defined as an estimated glomerular filtration rate (eGFR) <60 ml/min/1.73 m^2^] being monitored by a nephrologist and with no history of kidney transplantation or long-term dialysis. The patients were then followed up once a year for 5 years. The CKD-REIN study design and methods have been described in detail elsewhere (19, 20). The study protocol was approved by a national independent ethics committee (INSERM, Paris, France: reference: IRB00003888) and the French National Consultative Committee on Information Processing in Medical Research (*Comité Consultatif sur le Traitement de l’Information en matière de Recherche dans le domaine de la Santé*, Paris, France; reference: CCTIRS N° 12.360/CPP). All patients gave their written informed consent to participation.

### Data Collection

Data were collected extensively at baseline and then annually. Clinical data (including the CKD history, comorbidities, and medication use) were collected by trained clinical research associates from medical records and prescriptions. Data on sociodemographics, smoking habits, and dietician visits during the previous year were recorded either in patient interviews or via self-questionnaires. Blood pressure, height, and weight were measured. Patients were considered to have hypertension if this disorder was reported in their medical records or if they were taking antihypertensive medications. Patients were considered to have diabetes if (i) the disorder was self-reported, (ii) they used glucose-lowering medication, or (iii) they had a glycated haemoglobin level ≥6.5%, a fasting glucose level ≥7.0 mmol/L, or a randomly measured glucose level ≥11 mmol/L.

Each year, patients were prescribed a set of standard blood and urine tests to be performed at their usual clinical laboratory. Inflammation status was assessed with standard or ultrasensitive assays for serum CRP, depending on the assays used in that laboratory. The eGFR was estimated with the CKD Epidemiology Collaboration equation (21). Albuminuria or proteinuria were measured and classified according to the Kidney Disease: Improving Global Outcomes (KDIGO) 2012 categories: A1, normal (<3 mg/mmol); A2, moderately elevated (3–30); A3, severely elevated (>30) (20).

### Dietary Assessment

The patients self-administered a validated, short food frequency questionnaire (FFQ) once in 2017 (22). Each participant had to report his/her usual dietary intake over the previous year, the questionnaire included two parts. Part 1 of the FFQ focussed on intake frequencies and portion sizes during the previous year for 40 food items. Intake frequencies were quantified as “never or less than once a month,” “x times a month,” “x times a week,” or “x times a day.” Food portion sizes were estimated using standard serving sizes and food images. Part 2 of the FFQ focussed on the intake of nutrient-containing food items of specific interest in CKD, such as protein, sodium, and potassium. Based on these data, we calculated daily intakes in g per day for each food item by multiplying the intake frequency by the portion size. An *ad hoc* composition table was developed using data from the INCA2 survey (a representative survey of the general population in France), in order to estimate the percentage of each food included in a food item group (23). Nutritional data were then obtained using the food composition database developed by the French Data Centre on Food Quality (Ciqual, last updated in 2013) (24).

The FFQ also collected data on prebiotic and probiotic dietary supplements, ordinary yoghurts, and “probiotic yoghurts” (any yoghurt or yoghurt-like item containing strains like *Bifidobacteria*). The latter’s intake frequencies were qualified as daily or less than daily.

### Statistics

**FIGURE 1:**
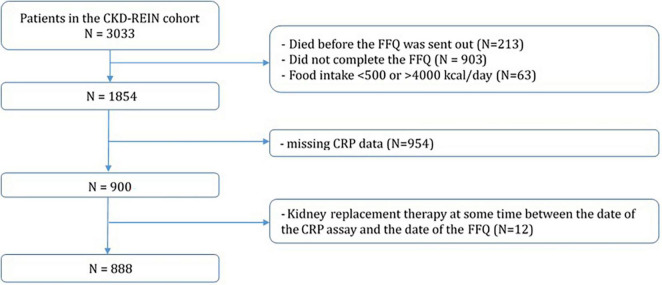
Study flowchart.

A total of 2,820 patients had completed the FFQ in 2017. We conducted a cross-sectional analysis of 888 (31%) patients who had CRP data for the year covered by the FFQ [i.e., blood samples taken in the 12 months preceding the FFQ completion date (Figure 1)]. The mean ± standard deviation time interval between inclusion in the CKD-REIN participants and completion of the FFQ was 1.96 ± 0.6 years. We first compared baseline characteristics for participants included in the present study and those not included. We then assessed participants as a function of their intake category (no yoghurt; ordinary yoghurt; probiotic yoghurt/dietary supplements), using an analysis of variance, a Kruskal–Wallis test or a chi-squared test, as appropriate. Next, we used logistic regression to estimate crude and adjusted odds ratio (ORs) and their 95% confidence intervals (CIs) for the association between inflammation and yoghurt or probiotic intake. Inflammation was studied as a binary variable, and the ORs were estimated for several commonly used CRP thresholds for inflammation (>3, >4, >5, >6, or >7 mg/L) (25–28). We checked for potential interactions between the CRP assay method (a standard assay for 59% of the participants, an ultrasensitive assay for 25%, and missing data for 16%) and yoghurt and probiotic intakes. Given that all the interactions were non-significant (i.e., *p* > 0.20 for all), we always adjusted the ORs for the assay method but did not include interaction terms in the models. We also adjusted for covariates significantly associated with inflammation in the crude analyses: age, educational level, atheromatous CVD, body mass index (BMI), CKD stage, and intakes of starch, eggs, and sweet snacks. We also forced variables known to affect inflammation (such as sex, and the protein, fibre, and total energy intakes) into the models. Since kidney replacement therapy (KRT) is likely to modify the relationship between yoghurt and probiotic intakes and inflammation, we removed the 70 patients having received KRT before the CRP assay date and FFQ completion from our sensitivity analyses (leaving 818 patients). Lastly, we investigated the association between inflammation and the frequency of yoghurt intake in five intake categories: no yoghurt consumption, occasional consumption of ordinary yoghurt, occasional consumption of probiotics, daily consumption of ordinary yoghurt and daily consumption of probiotics.

Due to the lack of data for some variables (missing data >5% for physical activity, albuminuria, and dietician visits), we applied multiple imputations (25 imputed datasets, fully conditional specification using all covariates, and a maximum of 30 iterations). Logistic regressions were fitted on each imputed dataset, and ORs, CIs and likelihood-ratio test *p*-values were combined according to Rubin’s rules (29); using the *mice* R package (30). All statistical analyses were performed using the R software (version 3.6.1) (31).

## Results

### Characteristics of the Study Population (Overall, and According to the Yoghurt and Probiotic Intake)

We included 888 participants (median age: 70; men: 65%; prevalence of diabetes: 40%), 164 (20%) of whom had consulted a dietician at least once (Table 1). The included and non-included CKD-REIN participants did not differ significantly with regard to clinical and demographic characteristics (Supplementary Table 1). The median [interquartile range (IQR)] eGFR was 29 [20–39] mL/min/1.73 m^2^. Thirty percent reported the consumption of probiotics (median [IQR] weekly intake: 7 [3–7] items), and 57% reported the consumption of ordinary yoghurts (median [IQR] weekly intake: 7 [3–8] items). When considering the patients who reported consuming probiotics, 84% consumed probiotic yoghurts only, 9% consumed probiotic dietary supplements only, and 7% consumed both. None of the participants reported the use of prebiotics alone as a dietary supplement.

**TABLE 1:** Characteristics of the study participants, overall and according to the intakes of ordinary yoghurt and probiotics.

	Overall	*N*	No yoghurt	Ordinary yoghurts	Probiotics	*P*-value
*N*	888		111	510	267	
**Demographic variables**						
Age (years)	70 [63–78]	888	73 [67–80]	70 [64–77]	70 [60–77]	0.029
Men (%)	576 (65)	888	77	66	58	0.003
Body mass index (kg/m^2^)	27 [24–31]	888	27 [25–30]	27 [24–31]	26 [24–31]	0.294
Smoking status (%)		888				0.051
Never smoker	355 (40)		38	37	46	
Former smoker	430 (48)		45	51	44	
Active smoker	103 (12)		17	11	10	
Educational level (%)		880				0.168
<9 years	547 (62)		67	64	57	
9–12 years	102 (12)		14	11	13	
≥12 years	231 (26)		19	26	30	
At least one consultation with a dietician (%)	164 (20)	834	10	21	20	0.027
Physical activity (1000 METs/min)	1.2 [0.2–4.2]	807	0.8 [0.0–2.9]	1.2 [0.2–4.3]	1.4 [0.4–4.2]	0.097
**Comorbidities**						
Diabetes (%)	350 (40)	887	44	42	32	0.009
Disease duration (years)	7.2 [4.6–12.7]	841	6.8 [4.9–11.2]	7.0 [4.4–11.8]	8.0 [5.0–14.6]	0.105
History of atheromatous CVD (%)	308 (36)	856	43	37	32	0.101
Hypertension (%)	805 (91)	883	86	92	92	0.082
**Kidney disease**						
eGFR (mL/min/1.73 m^2^)	29 [20–39]	888	30 [20–39]	28 [20–39]	29 [19–40]	0.789
CKD stage (%)		888				0.584
3	403 (45)		51	43	48	
4	349 (39)		37	41	36	
5 (not on dialysis)	66 (7)		6	7	9	
KRT	70 (8)		6	9	7	
Albuminuria (%)		825				0.188
A1	223 (27)		33	24	30	
A2	261 (32)		27	34	28	
A3	341 (41)		40	41	42	
**Diet**						
Protein (g/kg/d)	0.8 [0.6–1.1]	888	0.8 [0.6–1.0]	0.8 [0.6–1.1]	0.9 [0.6–1.2]	0.011
Fibres (g/d)	18 [13–23]	888	18 [12–24]	17 [12–23]	18 [13–24]	0.071
Sodium (g/d)	2.1 [1.5–2.8]	888	2.1 [1.6–2.7]	2.1 [1.5–2.9]	2.1 [1.5–2.8]	0.685
Total energy intake (kcal/d)	1663 [1264–2160]	888	1637 [1241–2119]	1663 [1261–2154]	1676 [1297–2194]	0.794
Egg intake (g/d)	14 [7–21]	888	14 [4–21]	14 [7–28]	14 [7–21]	0.022
Starches (g/d)	45 [23–68]	888	45 [22–68]	45 [23–68]	45 [23–70]	0.587
Sweet snacks (g/d)	23 [5–59]	888	23 [0–62]	20 [5–57]	28 [7–60]	0.215

*Data are quoted as the n (%) or the median [interquartile range]. CVD, cardiovascular disease; MET, metabolic equivalent task; CKD, chronic kidney disease; KRT, kidney replacement therapy; CRP, C-reactive protein.*

Relative to non-consumers, patients reporting ordinary yoghurt intake or probiotic intake were younger, more likely to be female and less likely to be active smokers (Table 1). They also had a higher level of physical activity and a higher protein intake and were more likely to have reported at least one consultation with a dietician visit. However, the consumers and non-consumers did not differ with regard to the eGFR level, the CKD stage or any of the clinical characteristics (with the exception of diabetes, which was less prevalent among participants reporting probiotic intake).

**FIGURE 2:**
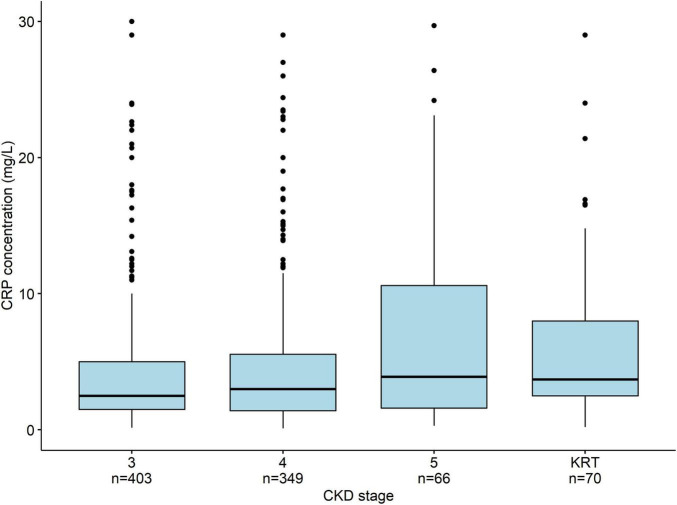
The median (IQR) serum CRP concentration as a function of the CKD stage. The CRP levels rise significantly (*p* < 0.001) with the CKD stage. To facilitate visual comparisons, outliers (including some >300 mg/L) are not displayed. The whiskers correspond to 1.5 times the IQR.

The median (IQR) serum CRP level in the overall population was 3.0 [1.6–7.0] mg/L. The CRP level increased significantly with the CKD stage (*p* < 0.001): 2.9 [1.5–6.0] for stage 3, 3.3 [1.5–6.7] for stage 4, 4.4 [2.0–13.0] for stage 5 not on dialysis, and 4.6 [2.5–9.0] mg/L for patients having received KRT (Figure 2).

### Association Between Inflammation and Yoghurt/Probiotic Intake

**FIGURE 3:**
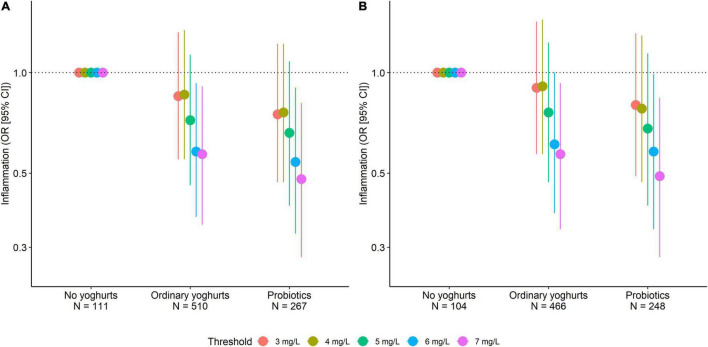
Adjusted odds ratios (95% CI) for various CRP thresholds as a function of intakes of ordinary yoghurt and probiotics **(A)** in all patients (*N* = 888) and **(B)** in patients not on KRT (*N* = 818). The adjustment variables included the CRP assay method, age, sex, educational level, BMI, history of atheromatous CVD, CKD stage, and intakes of starch, eggs, sweet snacks, protein, fibre, and energy.

The median CRP level and the proportion of people with a CRP level above 5 mg/L were significantly lower for participants consuming ordinary yoghurts and those consuming probiotics than for participants not consuming either (Table 2). Of note, CRP was significantly higher in patients with higher intakes of starches, eggs, sweet snacks, but was not associated with protein or fibre intakes (data not shown). After adjusting for confounders, the ORs for the associations between inflammation and ordinary yoghurt and probiotic intake were significantly lower for the CRP thresholds >6 mg/L and >7 mg/L (respectively, 0.58 [0.37, 0.93] and 0.57 [0.35, 0.91] for patients consuming ordinary yoghurts) and respectively, 0.54 [0.33, 0.9] and 0.48 [0.28, 0.81], for patients consuming probiotics (Figure 3A). This was not the case for the thresholds >3, >4, and >5 mg/L. Although the removal of the 70 patients with KRT from the analysis slightly attenuated these associations, the ORs for ordinary yoghurts and probiotics were still statistically significant for the CRP thresholds >6 mg/L and >7 mg/L (Figure 3B).

**TABLE 2:** Serum CRP concentrations and proportions of the values above the indicated thresholds, overall and according to the intakes of ordinary yoghurt and probiotics.

	Overall *N* = 888	No yoghurt *N* = 111	Ordinary yoghurts *N* = 510	Probiotics *N* = 267	*P*-value*
Median CRP level (mg/L)	3.0 [1.6, 7.0]	3.6 [2.2, 8.9]	3.1 [1.6, 7.0]	2.9 [1.5, 6.0]	0.036
CRP > 3 mg/L	49%	53%	50%	46%	0.315
CRP > 4 mg/L	41%	45%	42%	38%	0.317
CRP > 5 mg/L	33%	41%	34%	30%	0.164
CRP > 6 mg/L	28%	38%	28%	25%	0.036
CRP > 7 mg/L	25%	35%	25%	21%	0.017

*Intergroup comparisons of proportions were performed using a chi-squared test.

### Association Between Inflammation and the Frequency of Yoghurt/Probiotic Intake

Around half of the consumers of ordinary yoghurts and probiotics consumed these products daily (Supplementary Figure 1). With CRP thresholds >6 mg/L and >7 mg/L, the crude and adjusted ORs for the association with inflammation were significantly lower in all the consumer groups (relative to non-consumers) other than daily probiotic consumers (Supplementary Figure 2A). No dose-effect relationship was apparent. The exclusion of patients with KRT did not greatly affect these results (Supplementary Figure 2B).

## Discussion

Our cross-sectional analysis of patients with moderate-to-advanced CKD participating in the CKD-REIN cohort study showed that a substantial proportion consumed probiotics in yoghurts or dietary supplements. Our results also showed that both ordinary yoghurt intake and probiotic intake were associated with lower risk of inflammation when the CRP threshold was >6 mg/L or >7 mg/L. This association was independent of other known major determinants of inflammation but did not appear to be dose-dependent.

Some clinical trials have shown that probiotic supplementation is associated with a reduction in the level of inflammation in CKD patients. After 6 or 12 months of supplementation with probiotics or symbiotics, patients on dialysis showed a significant decrease in pro-inflammatory cytokines and CRP levels and an increase in anti-inflammatory cytokine levels (12, 15). In contrast, other clinical trials found a non-significant trend toward a lower CRP level (13, 14) or did not find any significant changes in inflammatory biomarker levels (16, 17, 32). These discrepancies might have been due to interstudy differences in the bacterial strains (sometimes combined with a prebiotic), intake doses, and intake duration (between 4 weeks and 6 months). Furthermore, the results of meta-analyses are inconclusive. Thongprayoon et al. (33) and Zheng et al. (34) found that probiotic or symbiotic intake was associated with a significant decrease in the CRP level, whereas Pisano et al. (35) and Tao et al. (36) did not find any significant differences. However, the clinical trials performed to date focused on small number of patients on dialysis, who are known to have higher levels of inflammation than healthy adults. The French calcium and phosphate observatory noted a median (IQR) CRP level of 5 (3–13) mg/L for haemodialysis patients (37), which is similar to the value observed for patients having received KRT in our study [4.6 (2.5–9) mg/L]. Low-grade inflammation is observed in early-stage CKD. We found that CRP level increased progressively with CKD stage; this is in line with the findings of epidemiological studies (38, 39), the Chronic Renal Insufficiency Cohort (1), and the Cardiovascular Health Study (40).

The only randomised study in this field investigated the relationship between probiotic use and CRP level over a 3-month period in CKD stage 3–4 patients. However, the sample size was low (*n* = 13) and the study lacked the power to demonstrate a significant effect (41). All the studies of probiotics were clinical trials that assessed the effect of dietary supplements but did not consider yoghurt intake. However, yoghurt is a probiotic by definition, since it contains the two regulated bacterial strains (*Lactobacillus delbrueckii* subsp. *bulgaricus* and *Streptococcus salivarius* subsp. *thermophilus*). The present study is the first to have investigated the “real-life” relationship between yoghurt/probiotic intake and inflammation in a CKD population. Interestingly, we found that yoghurt and probiotic intakes were both associated with lower inflammation when the latter was defined as a CRP threshold >6 mg/L. The removal of patients with KRT only slightly attenuated these associations. Our results are consistent with the body of evidence showing that (i) probiotics are associated with a reduction of inflammation in CKD patients (12, 15), (ii) the consumption of probiotic yoghurt or acidified milk reduces post-prandial inflammation after a high fat meal test in healthy subject (42), and (iii) the consumption of yoghurt is associated with lower levels of inflammation biomarkers in healthy adults (43, 44). In our study, however, the beneficial effect of yoghurt and probiotic consumption was only apparent for a high level of inflammation (CRP > 6 mg/L).

It is widely acknowledged that chronic, low-grade inflammation accelerates the progression of CKD, atherosclerosis and cardiovascular complications and increases the mortality rate (28, 45–47). Although CRP is widely assessed as a marker of systemic inflammation, the defining threshold varies from one study to another as a function of the health risks considered: it is typically 5 mg/L for mortality and 3 mg/L for CVD (28). In the present study, the association between yoghurt and probiotic consumption on one hand and the CRP level on the other was not statistically significant when low CRP thresholds (3, 4, and 5 mg/L) were considered – suggesting that the relationship was diluted for low-grade inflammation. Findings regarding the effect of yoghurt consumption on cardiovascular risk remain controversial. Meta-analyses of both observational studies and randomised clinical trials report inconsistent results, showing protective effects (48) or no effect (49) of yoghurt on cardiovascular risk or mortality. Recently, a meta-analysis showed that probiotic intake was associated with a decrease in blood pressure (50). Furthermore, frequent yoghurt and probiotic intake was linked to a lower level of albuminuria but not with CKD progression in the NHANES (18), and a meta-analysis did not observe a relationship between probiotic intake and eGFR preservation (36) – suggesting that a probiotic-based strategy has limited effects only.

Dysbiosis is one of the causes of chronic inflammation in CKD (51). This condition can be observed in early-stage CKD and can create a progressively pro-inflammatory environment in the host (2, 52). Dysbiosis in CKD patients results in the predominance of bacterial families that possess urease-, indole- and p-cresol-forming enzymes (53). The resulting uraemic toxins accumulate in the body fluids and contribute to inflammation (54). Dysbiosis also indirectly damages the epithelial tight junctions and increases gut barrier permeability; this allows bacterial products like lipopolysaccharides to leak into the circulation and contributes to inflammation. Shi et al. reported that the bacterial DNA concentration is positively correlated with plasma levels of CRP and IL-6 (52). It has been suggested that probiotics can modulate the composition of the microbiota (10), notably by competing with pathogens for nutrients and receptor binding sites (8, 55) and by decreasing levels of uraemic toxins (56). Probiotics also help to protect the intestinal barrier, reduce the activation of the pro-inflammatory nuclear factor-kappa B, and thus slow the leakage of lipopolysaccharides (8, 57). However, other dietary factors such as proteins and fibres may modulate gut microbiota and, potentially, the inflammation status (58, 59). In our study, these nutrients were not significantly associated with CRP level, in contrast with eggs, starches, and sweet snacks, and the observed association between yoghurt and probiotic intakes and inflammation status was independent of all these dietary factors. Moreover, González et al. (60) demonstrated that yoghurt had the best ability to modulate the faecal microbiota among all fermented dairy products.

Yoghurt and probiotics may limit low-grade inflammation. However, given that (i) dysbiosis is not the only source of inflammation in CKD patients (61), and (ii) yoghurt and probiotics mainly act on the gastrointestinal tract, the overall effect of consumption on inflammation in CKD patients may be limited. This limited effect was also suggested by our results for the frequency of consumption. We found that occasional yoghurt intake and daily yoghurt intake were equally associated with lower inflammation. This apparent lack of dose-dependence suggests that a certain quantity of bacteria (*Lactobacillus delbrueckii* subsp. *bulgaricus* and *Streptococcus salivarius* subsp. *thermophilus*) is enough to obtain a positive effect on the gastrointestinal tract and the microbiota. In contrast, occasional probiotic consumption (but not daily probiotic consumption) was associated with a significantly lower level of inflammation whatever the CRP threshold. However, this discrepancy might be due to a lack of statistical power because the ORs for CRP levels >6 mg/L and daily probiotic consumption were very similar to those for occasional or daily consumption of ordinary yoghurt.

The present study main strengths include its large population of CKD stage 3–5 patients (including a subgroup on KRT), the nationally representative network of investigating centres, and extensive data collection. Our detailed, validated FFQ enabled us to analyse ordinary and probiotic yoghurt consumption separately and to estimate the intakes of other nutrients (such as protein and fibre) that might modulate the composition of the microbiota. Our collection of clinical, nutritional and laboratory data enabled us to adjust our analyses for a large number of potential confounders, including relevant nutrients.

The study also had some limitations. Firstly, the study observational design prevented an assessment of the causal nature of the observed associations. Secondly, we only studied one inflammation biomarker. Nevertheless, CRP is acknowledged to be a reliable marker of inflammatory status (28, 62). Thirdly, the CRP data were generated in various local clinical laboratories, using standard or ultrasensitive assays. However, the type of assay used did not modify the studied associations. Fourthly, only 31% of the CKD-REIN participants alive in 2017 were eligible for our analysis; the remainder were excluded because they did not complete the FFQ, had inadequate food intake estimates, or did not have CRP assay data for the time period covered by the FFQ. Nonetheless, the participants included in or excluded from our analysis did not differ significantly with regard to their demographic and clinical characteristics. Lastly, recall bias cannot be ruled out when using FFQ, but ours has been validated against 24-hr recall over 1 year, and found to be reproducible and valid, thus limiting this bias (22).

## Conclusion

Our findings indicate that consumption of yoghurts and probiotics (regardless of the frequency of intake) is associated with a lower risk of inflammation in patients with CKD, independently of other known determinants of inflammation. Further research, including microbiota analysis, is needed to better understand the modulation of inflammation by yoghurts and probiotics and their potential impact on the progression and complications of CKD.

## Data Availability Statement

The data analysed in this study is subject to the following licenses/restrictions: Data available on request due to privacy/ethical restrictions. Requests to access these datasets should be directed to BS, benedicte.stengel@inserm.Fr.

## Ethics Statement

The study protocol was reviewed and approved by a National Independent Ethics Committee (INSERM, Paris, France: reference: IRB00003888) and the French National Consultative Committee on Information Processing in Medical Research (Comité Consultatif sur le Traitement de l’Information en matière de Recherche dans le domaine de la Santé, Paris, France; reference: CCTIRS N° 12.360/CPP). The patients provided their written informed consent to participate in this study.

## Author Contributions

SW, DF, ZM, and BS designed the present study. TM performed the statistical analyses. SW and BS drafted the manuscript. All authors were involved in the interpretation of the results and critical review of the manuscript and approved the manuscript as submitted.

## Conflict of Interest

SW received a grant from ISN-H4KH Initiative, outside the submitted work. LK received a grant from Fresenius Kabi outside the submitted work, together with consulting fees from AstraZeneca, Dr. Shäre, and Fresenius Kabi. DF received a grant from Fresenius Medical Care outside the submitted work, consulting fees from Fresenius Kabi and Sanofi, and honoraria from Lilly Fresenius Kabi, Sanofi, Vifor, and Astellas. ZM received honoraria from AstraZeneca and Boehringer Ingelheim. M-CB-R received honoraria from MAYOLY SPINDLER and GILEAD. The remaining authors declare that the research was conducted in the absence of any commercial or financial relationships that could be construed as a potential conflict of interest.

## Publisher’s Note

All claims expressed in this article are solely those of the authors and do not necessarily represent those of their affiliated organizations, or those of the publisher, the editors and the reviewers. Any product that may be evaluated in this article, or claim that may be made by its manufacturer, is not guaranteed or endorsed by the publisher.

## Abbreviations

BMI: body mass index
CI: confidence interval
CKD-REIN: CKD-Renal Epidemiology and Information Network
CVD: cardiovascular disease
eGFR: estimated glomerular filtration rate
FFQ: food frequency questionnaire
FAO: Food and Agriculture Organization
IL: interleukin
IQR: interquartile range
KDIGO: Kidney Disease: Improving Global Outcomes
KRT: Kidney replacement therapy
OR: odds ratio
NHANES: National Health and Nutrition Examination Survey
WHO: World Health Organization.
