# Supplementary material for: Probiotic Intake and Inflammation in Patients With Chronic Kidney Disease: An Analysis of the CKD-REIN Cohort
Source: Front Nutr. 2022 Mar 30;9:772596. doi: 10.3389/fnut.2022.772596 (PMC9005823; doi:10.3389/fnut.2022.772596)
Supplement: Supplementary file 1 [file Table_1.DOCX]

SUPPLEMENTARY MATERIALS

Supplementary Table 1: Characteristics of study participants and non-participants.

|  | Overall | N | Non-participants | Participants | p |
| --- | --- | --- | --- | --- | --- |
| N | 2820 |  | 1932 | 888 |  |
| Age at baseline (years) | 68 [60, 76] | 2820 | 68 [59, 76] | 68 [61, 76] | 0.193 |
| Men | 1829 (65) | 2820 | 64 | 66 | 0.309 |
| Educational level |  | 2789 |  |  | 0.587 |
| <9 years | 1770 (64) |  | 64 | 62 |  |
| 9–12 years | 319 (11) |  | 11 | 12 |  |
| ≥12 years | 700 (25) |  | 25 | 26 |  |
| Smoking status |  | 2820 |  |  | 0.249 |
| Never smoker | 1186 (42) |  | 43 | 40 |  |
| Former smoker | 1302 (46) |  | 45 | 48 |  |
| Active smoker | 332 (12) |  | 12 | 12 |  |
| At least one consultation with a dietician | 604 (22) | 2737 | 21 | 24 | 0.138 |
| Physical activity (1000 METs/min) | 1.1 [0.1, 3.6] | 2362 | 1.1 [0.0, 3.4] | 1.2 [0.2, 4.2] | 0.044 |
| Diabetes | 1184 (42) | 2814 | 43 | 39 | 0.059 |
| Disease duration (years) | 5.1 [2.5, 10.3] | 2686 | 5.1 [2.5, 10.1] | 5.2 [2.4, 10.5] | 0.548 |
| History of atheromatous CVD | 959 (35) | 2757 | 34 | 36 | 0.388 |
| Hypertension | 2554 (91) | 2813 | 91 | 91 | 0.674 |
| eGFR (mL/min/1.73m²) | 32 [24, 42] | 2820 | 33 [24, 42] | 31 [23, 41] | 0.062 |
| Albuminuria |  | 2507 |  |  | 0.505 |
| A1 | 711 (28) |  | 29 | 27 |  |
| A2 | 795 (32) |  | 32 | 32 |  |
| A3 | 1001 (40) |  | 39 | 41 |  |

Supplementary Table 2: Serum CRP concentrations and proportions of the values above the indicated thresholds, overall and according to the frequency of intake of ordinary yoghurt and probiotics

|  | Overall  N=888 | No yoghurt  N = 111 | Ordinary yoghurts (occasional)  N = 271 | Probiotics (occasional)  N = 89 | Ordinary yoghurts (daily)  N = 301 | Probiotics (daily)  N = 116 | P-value |
| --- | --- | --- | --- | --- | --- | --- | --- |
| Median CRP (mg/L) | 3.0 [1.6 , 7.0] | 3.6 [2.2, 8.9] | 3.1 [1.5 , 7.0] | 2.5 [1.2 , 5.0] | 3.0 [1.6 , 6.8] | 3.1 [1.6 , 7.8] | 0.068 |
| CRP >3 mg/L | 49% | 53% | 51% | 37% | 50% | 50% | 0.175 |
| CRP >4 mg/L | 41% | 45% | 42% | 28% | 41% | 44% | 0.107 |
| CRP >5 mg/L | 33% | 41% | 33% | 25% | 33% | 36% | 0.194 |
| CRP >6 mg/L | 28% | 38% | 27% | 20% | 27% | 30% | 0.073 |
| CRP >7 mg/L | 25% | 35% | 24% | 17% | 24% | 26% | 0.054 |

Legend of supplementary figures:

Supplementary Figure 1: Frequency of intake (daily or occasional) of yoghurts and probiotics by the study participants.

Supplementary Figure 2. Adjusted odds ratio (with their 95% confidence intervals) for inflammation, being above a specific CRP concentration, according to the frequency of yoghurts and probiotics intake, for A) all patients and B) for patients never having received RRT (N = 818). Adjustment variables were CRP quantification method, age, educational level, BMI, a history of atheromatous CVD, CKD stage, and intake of starch, eggs and sweet snacks and also for sex and protein, fibre and energy intakes.
