# Supplementary figures and images for: Probiotic Intake and Inflammation in Patients With Chronic Kidney Disease: An Analysis of the CKD-REIN Cohort
Source: Front Nutr. 2022 Mar 30;9:772596. doi: 10.3389/fnut.2022.772596 (PMC9005823; doi:10.3389/fnut.2022.772596)

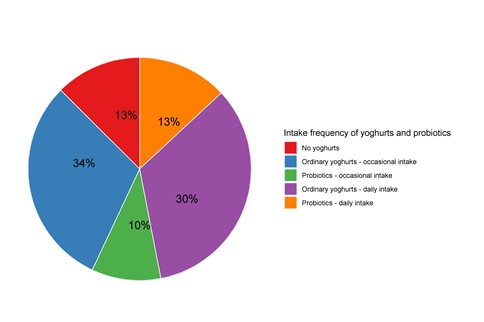

Supplement: Supplementary file 2 [file Image_1.jpeg]

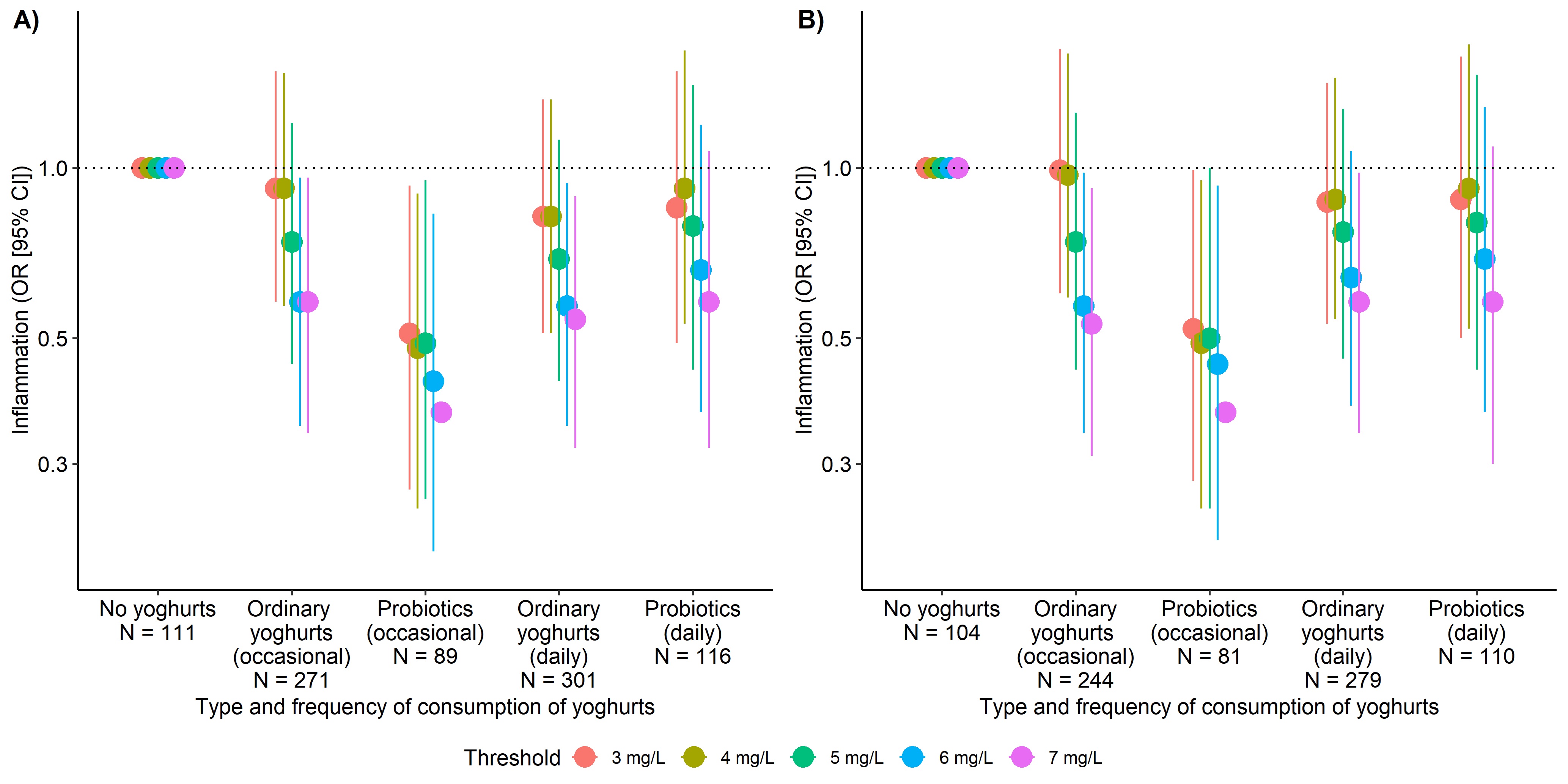

Supplement: Supplementary file 3 [file Image_2.jpg]
